# Physics Constrained High-Precision Data-Driven Modeling for Multi-Path Ultrasonic Flow Meter in Natural Gas Measurement

**DOI:** 10.3390/s24144521

**Published:** 2024-07-12

**Authors:** Haohui Cai, Wensi Liu, Kaixi Zhou, Xin Wang, Kunwei Lin, Xiao-Yu Tang

**Affiliations:** 1 PipeChina West Pipeline Company Ltd., Xinjiang 830013, China; caihh@pipechina.com.cn; 2 Xinjiang Key Laboratory of Multi-Medium Pipeline Safety Transportation, Xinjiang 830013, China; 3 College of Control Science and Engineering, Zhejiang University, Hangzhou 310027, China; ws.liu@zju.edu.cn (W.L.); 3200101468@zju.edu.cn (K.Z.); 22032087@zju.edu.cn (X.W.); 22032090@zju.edu.cn (K.L.)

**Keywords:** ultrasonic flow meter, mechanism knowledge, data-driven model, prediction model

## Abstract

Ultrasonic flow meters are crucial measuring instruments in natural gas transportation pipeline scenarios. The collected flow velocity data, along with the operational conditions data, are vital for the analysis of the metering performance of ultrasonic flow meters and analysis of the flow process. In practical applications, high requirements are placed on the modeling accuracy of ultrasonic flow meters. In response, this paper proposes an ultrasonic flow meter modeling method based on a combination of data learning and industrial physics knowledge. This paper builds ultrasonic flow meter flow velocity prediction models under different working conditions, combining pipeline flow field velocity distribution knowledge for data preprocessing and loss function design. By making full use of the characteristics of the physics and data learning, the prediction results are close to the real acoustic path flow velocity distribution; thus, the model has high accuracy and interpretability. Experiments are conducted to prove that the prediction error of the proposed method can be controlled within 1%, which can meet the needs of ultrasonic flow meter modeling and subsequent performance analysis in actual production.

## 1. Introduction

With the increasing emphasis on environmental protection in society, the energy structure is undergoing a transition from the predominant use of traditional coal and oil toward a more diverse and cleaner direction. Compared to other fossil fuels, burning natural gas produces less carbon dioxide and other pollutants. Natural gas, regarded as a relatively clean fuel, is widely applied in sectors such as electricity, industry, and transportation [1]. It is considered a crucial component for achieving the transformation of the energy structure and reducing carbon footprints.

The majority of metered trade in gaseous natural gas is conducted through pipelines. The accurate measurement of natural gas flow is key to ensuring energy management, metering and settlement, and pipeline transportation safety. Natural gas is often transported at high pressures and temperatures, which makes it difficult to calibrate and operate flow measurement equipment. In addition, in actual pipelines, natural gas may have multi-phase flow conditions of liquid, gas and solid, which increases the complexity of flow measurement. Traditional flow meters such as orifice plate flow meters [2] and turbine flow meters [3] have played a crucial role in the metering of transported natural gas. In recent years, ultrasonic flow meters [4] have gained widespread use due to their advantages such as no blocking devices, high repeatability, wide measurement range, simple operation, bidirectional measurement, high precision and easy installation. So far, multiple countries, including the United States, the United Kingdom, Germany, and the Netherlands, have designated ultrasonic flow meters as legal measuring instruments for natural gas trade transactions [5]. In China, ultrasonic flow meters have also been applied in pipeline flow metering within projects like the “West–East Gas Transmission”. In 2020, China had already deployed over 3000 units of gas ultrasonic flow meters in metering operations, establishing themselves as crucial instruments in the field of natural gas metering and trade [6].

**Figure 1:**
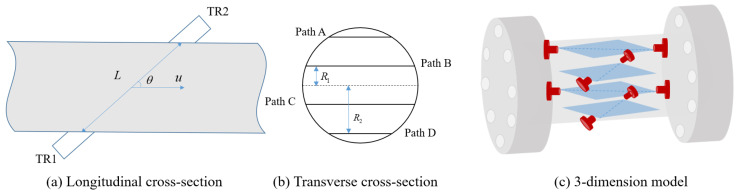
Multi-path ultrasonic flow meter (four paths) made by DANIEL.

The flowing gas medium modulates the propagated ultrasonic signals, manifesting in physical characteristics such as the speed, frequency, and acoustic energy of the sound waves, which carry information related to the gas flow velocity. Detecting the gas flow velocity information contained in these physical characteristics and employing mathematical methods to convert it into gas flow information enable the measurement of gas flow. Taking the time difference method ultrasonic flow meter [7,8,9] (Figure 1a) as an example, it measures the flow by determining the time difference between the forward and reverse propagation of ultrasonic pulses. Ultrasonic transducers TR1 and TR2 are fixedly mounted upstream and downstream on the flow meter body. Upstream transducer TR1 emits ultrasonic signals, and downstream transducer TR2 receives the ultrasonic signals, marking the propagation time of the ultrasonic signals from TR1 to TR2 as the forward propagation time ($t_{down}$). Downstream transducer TR2 emits ultrasonic signals, and upstream transducer TR1 receives the ultrasonic signals, marking the propagation time of the ultrasonic signals from TR2 to TR1 as the reverse propagation time ($t_{up}$).

The forward propagation time is expressed in Equation (1).

(1) $$t_{down}=\frac{L}{c+u\cos\theta }.$$

And the reverse propagation time is represented in Equation (2),

(2) $$t_{up}=\frac{L}{c-u\cos\theta }$$

where *u* represents the average velocity of the acoustic channel line, *c* is the speed of sound propagation, *L* is the distance between the two ultrasonic transducers, and θ is the angle between the line connecting the two ultrasonic pulses and the axial direction. It should be further explained that the acoustic channel refers to the path through which the ultrasonic signal is transmitted in the flow meter.

By subtracting the two equations above, the speed of sound *c* can be eliminated, and thus, the average fluid velocity on the acoustic channel *u* can be obtained by Equation (3).

(3) $$u=\frac{L}{2\cos\theta }\left(\frac{1}{t_{down}}-\frac{1}{t_{up}}\right)$$

When fluid flows in a long straight circular pipe, if the effect of fluid viscosity is ignored, the velocity at each cross-section of the pipe should be uniformly distributed. Due to the absence of friction, there is no pressure loss along the direction of flow in the pipe. However, the actual viscosity of the fluid will result in an uneven distribution of velocity across the pipe cross-section. Specifically, the velocity decreases closer to the pipe wall, becomes zero at the wall, and reaches its maximum value at the central axis according to Newton’s law of viscosity [10]. At the same time, frictional losses cause a reduction in pressure along the direction of flow in the pipe.

Different brands of flow meters employ distinct arrangements for their acoustic paths. The acoustic path configurations for DANIEL are depicted in Figure 1b,c. Channels A, D and B, C of DANIEL are equidistant from the pipeline center axis, measuring $R_{2}$ and $R_{1}$, respectively ($R_{1}$ and $R_{2}$ are constants given by the manual) [11].

The velocity data collected by ultrasonic flow meters, along with the corresponding operational condition data, together constitute a set of sample data. These sample data are crucial for the metrological performance analysis and flow process analysis. Through modeling, the flow and transmission characteristics of natural gas under different conditions can be deeply understood, thereby optimizing the design and calibration methods of flow meters and improving the metering accuracy. How to use sample data to establish a high-precision model of ultrasonic flow meter is an important research topic. In natural gas application scenarios, the main difficulty in flow meter modeling is to deal with non-ideal gas behavior, multi-phase flow phenomena and dynamic changes in flow states under high pressure and high-temperature conditions of natural gas. These factors place extremely high requirements on the accuracy, real-time correction capability and adaptability of the model.

On the one hand, the modeling methods based on physics knowledge have been widely used in the modeling of ultrasonic flow meters. Computational Fluid Dynamics (CFD) [12,13], as an important engineering technology, plays a vital role in the field of fluid mechanics. Roman et al. [14] deal with CFD simulation and conducted an experimental study of ultrasonic flow meters. A mathematical model of an ultrasonic flow meter is built for studying the errors of flow measurement in disturbed flows. The method of defining the position coordinates of the acoustic paths and their weighting factors is improved based on the Gauss–Jacobi method of integration, which provids the possibility of raising the accuracy of the turbulent flow velocity integration. The Navier–Stokes equation is a basic equation in fluid mechanics and is used to describe the motion of viscous fluids. In CAGF [15], three-dimensional, unsteady, compressible Navier–Stokes equations [16] are solved by a finite volume scheme, which is based upon the second-order upwind scheme for spatial derivatives and the multi-stage Runge–Kutta integral method for time derivatives. In order to simulate the multi-path ultrasonic flow meter, an excited pressure signal is applied to three different locations upstream, and the pressure signals are received at three different locations downstream. Finally, the simulation results are analyzed and visualized through postprocessing techniques. Li et al. [17] establishes a mathematical model for a multi-path ultrasonic flow meter based on the principle of time-of-flight ultrasonic flow measurement and the Gauss–Legendre numerical integration method. In the modeling process, based on the formula for integrating the instantaneous flow velocity with the flow velocity distribution function over the area, the formula for the instantaneous flow velocity weighted sum of the average flow velocity at the chord-wise channels is derived. Legendre polynomials are applied to solve for Gauss nodes and weighting coefficients, determining the distribution positions of each path. In summary, the mechanistic modeling approach involves the application of various numerical methods, grid generation algorithms, and high-performance computing techniques. The advantages of these methods lie in their ability to provide a deep understanding and accurate description of fluid behavior, aiding in the revelation of the internal physical mechanisms and regularities of the system. Moreover, they offer effective tools for engineering design. However, mechanistic modeling also has some drawbacks, such as high demands on computational resources, the significant impact of model parameter selection on the results, and potential numerical instability in certain cases. Additionally, the accuracy of the model depends on the precise understanding of fluid behavior and boundary conditions, leading to possible errors and uncertainties.

On the other hand, with the development of deep learning technology, data-driven modeling methods [18,19,20] have been able to sufficiently solve problems in different production and life aspects. In NER [21], a multi-layer perceptron neural network-based calibration is proposed for a utility ultrasonic flow meter. Since calibration equations is a mapping between the flow meter and prover or master meter reading, this article makes full use of the nonlinear mapping capabilities of neural networks to predict the flow rate with a smaller error. Li et al. [22] uses the random forest algorithm to predict the flow deviation of the ultrasonic flow meter in use on the basis of obtaining the signal quality data, flow pattern index data and metering performance data of the ultrasonic flowmeter. Li et al. [23] establish a multi-acoustic path line-averaged velocity correction model based on the BP neural network for the ultrasonic flow meter, aiming to explore the relationship between the directly measured line-averaged velocity and the cross-sectional average velocity of the pipe. These methods excel in utilizing a large volume of actual measurement data for modeling, providing highly accurate flow predictions or corrections, which are particularly suitable for complex fluid environments and pipeline structures. Data-driven approaches do not rely on specific physical models or assumptions, thus offering strong flexibility and adaptability, and performing exceptionally well in scenarios with high real-time requirements. However, data-driven modeling also presents challenges and limitations, such as a dependence on large quantities of high-quality data and the black-box nature of the models, making internal structures difficult to interpret.

To sum up, in the ultrasonic flowmeter modeling process, the mechanism modeling has the problem of insufficient accuracy because it cannot fully capture the complex fluid behavior and the influence of multiple factors. Although data-based modeling can handle complex nonlinear problems, its black box nature leads to poor interpretability and difficulty in providing clear physical meaning. In order to solve these problems, this paper proposes an ultrasonic flow meter modeling method based on a combination of data learning and industrial background. Fully mining the distribution information of real data and using physical laws as constraints can improve the interpretability and accuracy of data-driven modeling. In this study, we delve into the designing of flow rate prediction models for ultrasonic flow meters across diverse operating conditions. Mechanistic knowledge is integrated to preprocess the input data, which includes operations such as normalizing continuous data, encoding discrete data, and converting the base of multiple binary discrete data. We construct a multi-layer perceptron network, integrating both the relative error and the constraints imposed by mechanistic principles on pipeline flow velocity distribution into a unified loss function. Following training, the model effectively predicts flow velocity, thus fulfilling the objective of modeling for ultrasonic flow meters.

In sum, our contributions are three-fold:

- We propose an ultrasonic flow meter modeling method based on a combination of data learning and industrial physics knowledge. The proposed method overcomes the disadvantages of single modeling and shows high accuracy and interpretability.
- In terms of data preprocessing, different processing methods are adopted for different data based on the data type and the degree of impact on the flow process. This personalized data preprocessing method helps maximize the extraction of useful information and reduce the impact of noise and irrelevant variables.
- In terms of loss function design, the distribution of the real acoustic path flow velocity is added as a limiting mechanism to the loss function, which can make the prediction of the model as close as possible to the real flow velocity.

The rest of this article is organized as follows. Section 2 provides an overview of the proposed modeling method for our ultrasonic flow meter, including data preprocessing and model details. Section 3 illustrates and discusses the results of the proposed model. Section 4 concludes this article.

## 2. Methodology

**Figure 2:**
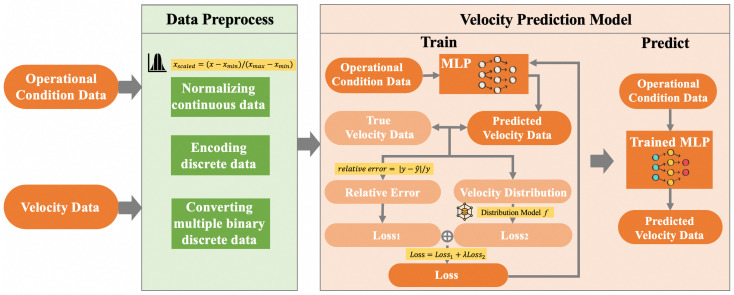
The overview of the proposed method.

In Figure 2, the process diagram of the proposed method is depicted. Based on the knowledge of flow-related mechanisms, we selected operating condition data that influence the flow velocity, collectively forming an operating condition dataset. Then, we integrated the mechanistic knowledge into the preprocessing of input data, including operations such as normalizing continuous data, encoding discrete data, and converting the base of multiple binary discrete data. Subsequently, we constructed a predictive model for ultrasound flow meter velocity, using the preprocessed operational condition data as input and acoustic path velocities as output. The true velocity data are used as the label. By analyzing the label, we can obtain the true flow velocity distribution. The model training process is supervised by a joint loss function that combines the relative error and the constraints imposed by the mechanism of flow velocity distribution. Through this innovative modeling approach, we can achieve the precise prediction of velocity for each acoustic path under the specified working condition data in the prediction phase.

### 2.1. Data Preprocessing

During preprocessing, we first select variables that have a significant impact on the flow process as input data based on expert experience. These variables include the pipe diameter, temperature, pressure, rectifier, collector pipe condition, distance before rectification, distance before flow meter and standard flow rate. The input working condition data have different dimensional units and scale ranges. Through data preprocessing operations, the original data can be made cleaner, standardized and suitable for model use. Here, we combine data types and flow-related mechanism knowledge to adopt different processing methods for different data.

Specifically, continuous data in the input are subjected to min–max normalization [24], mapping it to the range [0, 1]. Min–max normalization eliminates the influence of dimensions, mapping all feature values to the same interval, thereby eliminating differences and ensuring a more balanced impact of all features on the model. According to the knowledge of flow-related mechanisms, the standard flow rate has the greatest impact on the velocities of the various acoustic paths of the ultrasonic flow meter. Therefore, mapping the standard flow rate to a larger interval can amplify its influence on the model. To be specific, we normalized continuous data, such as the pipe diameter, temperature and pressure, to the range of [0, 1], and we normalized the standard flow rate to [0, 10].

For discrete data in the input, each value is assigned an integer label, which is mapped to a relatively uniform integer space. Then, through min–max normalization, the integer labels are mapped to the range [0, 1], further standardizing the data scale and eliminating differences in the original integer labels. In our scenario, the rectifier status, distance before rectification and distance before the flow meter are discretely coded and normalized.

For the multiple binary discrete data in the input data, we treat them as multi-bit binary numbers and process them as one feature. We converted the multi-bit binary numbers to decimal numbers and then performed the described min–max normalization on them, mapping them to the range [0, 1]. In the working condition data, the inflow situations at the 3rd, 4th, 5th, 6th, 7th and 8th entrances are collectively referred to as the collector pipe situation. The values for each entrance are discrete 0 or 1. Treating the inflow situations of entrances 3rd–8th as a single feature not only aligns with the practical meaning of representing the collector pipe inflow situation but also reduces the dimensionality of the input data, preventing from occupying too many input dimensions and ensuring a more balanced impact of all features on the model. Converting multi-bit binary numbers to decimal numbers simplifies the representation of features, consolidating information from multiple binary bits into a single numerical value. This aids in reducing dimensions, improving model training efficiency, and better adapting to machine learning algorithms.

In conclusion, we map all feature variables (except the standard flow rate) to intervals of the same scale through min–max normalization and discrete data encoding, thereby eliminating scale range differences and ensuring a more balanced impact of all features on the model. In addition, the standard flow rate has the greatest impact on the velocities of the acoustic paths of the ultrasonic flow meter. We can amplify its impact on the model by normalizing the standard flow rate to a larger interval. These personalized data preprocessing methods can clean and prepare data more effectively, better retain the characteristic information of the data, and accelerate the model training process. In addition, based on the mechanistic knowledge of the flow process, appropriate processing methods can better reflect the actual scenario and improve the model’s understanding and prediction ability of the flow process. This provides more reliable support for predictions.

### 2.2. Velocity Prediction Model

The ultrasonic flow meter flow velocity prediction model uses MLP (Section 2.2.1) as the network architecture, which establishes a relationship model between the operating condition data and acoustic path flow velocity. In the training phase of the network, on the basis of obtaining predicted and real flow velocity data, the training of the model is supervised through a carefully designed loss function (Section 2.2.2). After training, MLP has great nonlinear modeling capability. By inputting operating condition data into the model, high-precision flow velocity data can be obtained, which can achieve the purpose of flow velocity prediction.

#### 2.2.1. Multi-Layer Perceptron

Multi-layer perceptron (MLP) [25] is a type of feedforward artificial neural network that consists of at least three layers: an input layer, hidden layers, and an output layer. Except for the input nodes, each node is a neuron that uses a nonlinear activation function [26]. MLP is trained through forward propagation and back-propagation. Forward propagation is used to calculate the output, and the performance of the model is measured through the loss function. Back-propagation is used to update the weights of the model to minimize the loss function. MLP has wide applications in many fields, including image recognition, natural language processing, recommendation systems, etc.

Here, we use the MLP network, taking the preprocessed working condition data as input and the flow velocity of each acoustic path as output, to build an ultrasonic flow meter flow velocity prediction model.

#### 2.2.2. Loss Function Design

Loss function is a concept widely used in machine learning and optimization problems. It measures the error between model predictions and actual observations. During the training process, the parameters of the model are adjusted by minimizing the loss function, allowing the model to predict the target variable more accurately.

In the flow velocity prediction model of the ultrasonic flow meter, our loss function consists of two parts. Firstly, we input the preprocessed operating condition data into an MLP network to obtain the predicted velocity. The relative error between the predicted value and the actual value is used as the first part of the loss function, which is denoted as *loss*_1_. Using the relative error as the loss function helps the model be more robust when dealing with true values in different ranges.

**Figure 3:**
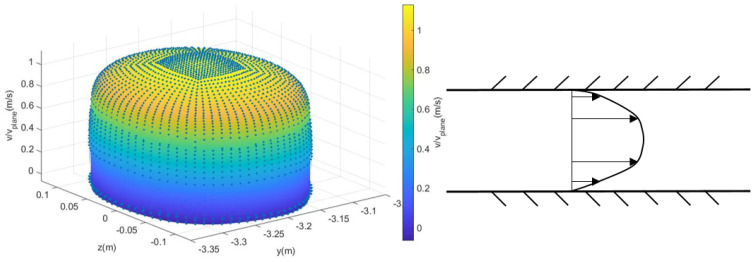
Three-dimensional flow velocity profile and its longitudinal profile.

Secondly, we consider adding the real flow field distribution to the loss function as a mechanism constraint. In pipelines, when flow is fully developed, the shape of the velocity profile (Figure 3) is influenced by the pipe diameter. It has been observed that as the pipe diameter increases, the velocity profile becomes flatter [27]. In large-diameter pipelines, fluid flow is relatively stable. The impact of the pipe wall on fluid friction is minimal, facilitating a more uniform distribution of mass and momentum in the fluid and resulting in a relatively smooth velocity profile. In contrast, in small-diameter pipelines, due to the relatively constrained flow space, the flow is more susceptible to the influence of wall friction, leading to more pronounced variations in the velocity profile and a steeper appearance.

In terms of data [27], as the pipe diameter decreases, the flow velocity profile becomes sharper, meaning the ratio of the flow velocity in the sound channel near the diameter to that in the other sound channels increases. Conversely, when the pipe diameter is larger, the flow velocity profile is blunter, meaning the ratio of the flow velocity in the sound channel near the diameter to that in others is smaller.

Based on this observation, the flow velocity distribution model *f* of the ultrasonic flow meter under different diameters can be established, and it can be added to the data-driven model as a mechanism restriction. Specifically, it can be used as the loss function *loss*_2_ (Equation (4)). In detail, when determining the reference velocity profile *f*, we first collect existing actual acoustic path flow velocity data and discuss it based on different diameters. Next, we determine the parameters of the power-law model [28] and formulate the flow curve equation. For a given diameter, we use all flow velocity data under this diameter to fit a velocity curve with the highest accuracy, which serves as our reference velocity profile *f*. The flow velocity prediction model will generate the predicted flow velocity of each acoustic path for the operating condition data. For each set of flow velocity prediction data, we can obtain its velocity curve $\hat{f}$ through power-law model and fitting. If a distribution $\hat{f}$ described by a certain set of flow velocities does not conform to the flow velocity distribution law described by *f*, then a large *loss*_2_ will penalize the prediction model. On the contrary, if it conforms to the flow velocity distribution represented by *f*, the value of *loss*_2_ will become smaller. Adding mechanism constraints to the loss function allows the distribution of flow velocity in the predicted values to be supervised and trained so that the model’s predictions are as close as possible to the true flow velocity distribution. It not only conforms to the mechanism characteristics of real flow data under the same pipe diameter but also ensures good linearity between the predicted values under different input data, exhibiting great accuracy.

(4) $$loss_{2}=\int_{-R}^{R}\left|f-\hat{f}\right|.$$

The relative error and the mechanism limitations on pipeline flow velocity distribution rule are jointly used as the loss by Equation (5),

(5) $$loss=loss_{1}+\lambda loss_{2}$$

where λ is the weight value, which is used to balance the impact of the two losses.

## 3. Experiment

### 3.1. Experiment Setup

**Figure 4:**
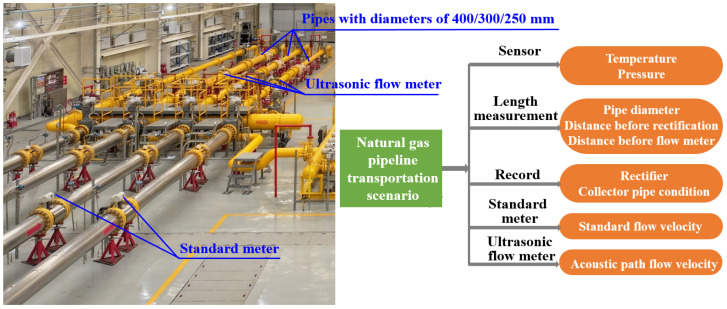
Actual measurement diagram and test process.

The operational condition data and corresponding flow rate data together constitute the dataset required for modeling. We selected variables such as the pipe diameter, temperature, pressure, rectifier, collector pipe condition, distance before rectification, distance before flow meter, standard flow rate and other parameters related to flow conditions as operational condition data. In our experiments, the specific value ranges or definitions of these variables can be obtained in Table 1. Figure 4 shows an applied structure image of actual measurements. The ultrasonic flow meter is installed after the rectifier. In addition, the installation point should be sufficiently far away from interference sources such as valves, pumps, and frequency converters. The information related to the temperature and pressure is obtained through the sensor, and the information related to the pipe diameter, distance before rectification, and distance before the flow meter can be obtained through length measurement. Rectifier and collector pipe conditions can be logged directly. Through the standard meter, we can obtain the standard flow rate data, and the acoustic path flow velocity can be read with an ultrasonic flow meter.

### 3.2. Experiment Result

In the data preprocessing stage, we normalized the continuous data, such as the pipe diameter, temperature and pressure to the range of [0, 1], and we normalized the standard flow rate to [0, 10]. The rectifier status, distance before rectification and distance before flow meter were discretely coded and normalized. The inflow situations at the 3rd, 4th, 5th, 6th, 7th, and 8th entrances were uniformly treated as the collector pipe condition. We converted the multi-digit binary in this case to a decimal and then normalized it. In the ultrasonic flow meter flow velocity prediction model, the preprocessed operational condition data are considered as the input data, and the flow velocity data of each channel collected by the ultrasonic flow meter are the output data.

(6) $$MAPE=\frac{1}{n}\sum_{i=1}^{n}\frac{|v_{true}-v_{pred}|}{v_{true}}\times 100\%.$$

**Figure 5:**
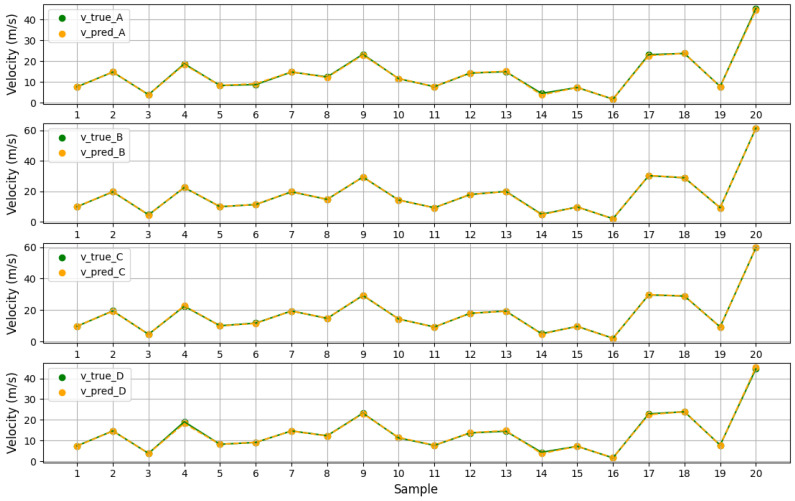
Flow velocity data of four paths on the test set.

**Figure 6:**
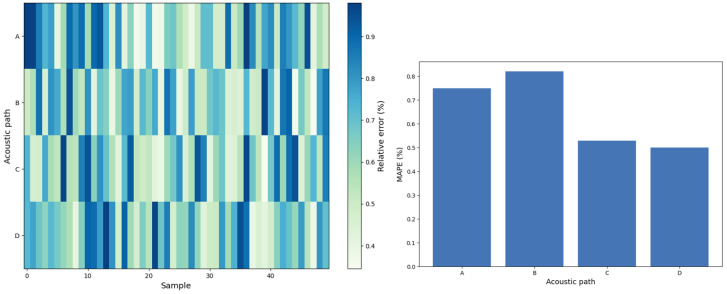
Relative error on the test set.

We utilized an MLP network with two hidden layers as the prediction model. The number of neurons in the hidden layers were 50 and 30, respectively, with Rectified Linear Unit (ReLU) activation functions. The number of neurons in the input and output layers was determined by the dataset. We collected 250 pieces of data from actual application scenarios. Through random sampling, 80% of the data were selected for training and 20% of the data were used for testing. We first took 20 groups of samples from the test set and displayed the predicted flow velocity and the real flow velocity in Figure 5. We found that the two kinds of flow rate data points basically coincide. Then, we compared the predicted values with the actual flow rate data and calculated the relative error. The Mean Absolute Percentage Error (MPAE) can be obtained from the relative error (Equation (6)). The errors of five representative pieces of data are shown in Table 2. Among these prediction results, the smallest relative error is close to 0, and the largest relative error is only 0.96%. Figure 6 shows the relative error of each acoustic path on all test data (left) and the MAPE of each acoustic path (right). Moreover, through calculation and statistics, the MAPE of all data is 0.65%. The small MAPE value demonstrates the high accuracy of our method.

**Figure 7:**
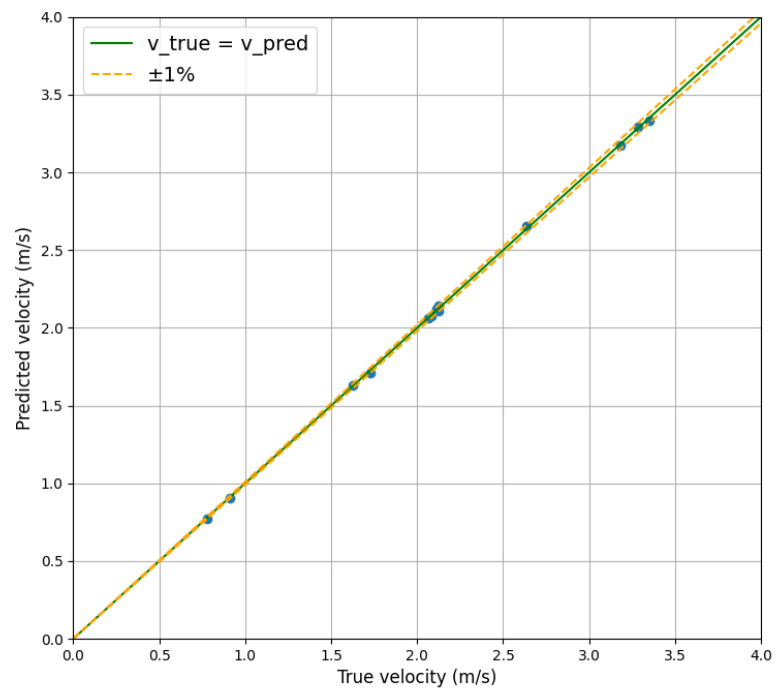
Display of relative error. The velocity values range from 0 to 4 m/s. Blue dots are samples.

**Figure 8:**
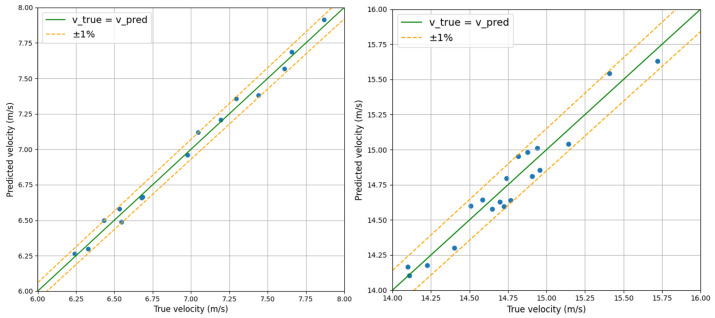
Magnified display of relative error. The flow velocity is around 7 m/s and 15 m/s.

We extracted some flow velocity data within the range of 0 to 4 m/s, comparing the predicted velocity with the actual velocity, as shown in Figure 7. The graph plots the actual velocity on the horizontal axis and the predicted velocity on the vertical axis with a green line representing the velocity values when the predicted values match the actual values. The orange dotted lines are the error lines of ±1%. Our flow velocity data (blue dots) are distributed around the green line, which visually demonstrates that our method is capable of generating high-precision predicted velocities. To illustrate the relative error more clearly, we plot the values of actual and predicted velocities near 7 m/s and 15 m/s in Figure 8. Observing Figure 8, it can be noted that the data points are distributed relatively evenly on both sides of the green line. When the data point is above the green line, it indicates that the predicted velocity is higher than the actual velocity. Conversely, when the data point is below the green line, the opposite is true. However, all data points are within the range of the orange dotted lines, indicating that the relative error calculated by our method will not exceed 1%.

### 3.3. Ablation Study

**Figure 9:**
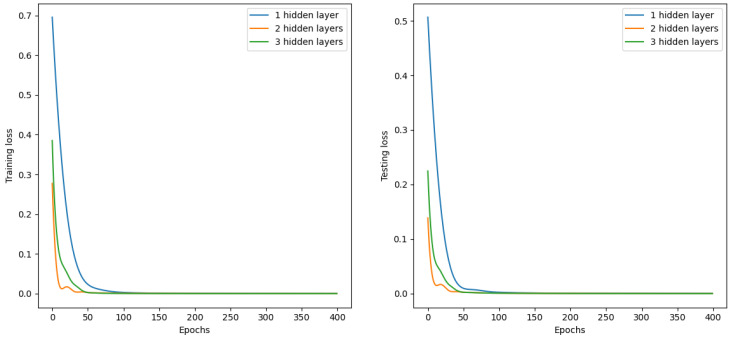
Training and testing loss curves.

To demonstrate the effectiveness of model selection, we conducted an ablation study on the scale of the MLP network. We experimented with network architectures consisting of one hidden layer (with 50 neurons), two hidden layers (with 50 and 30 neurons), and three hidden layers (with 50, 30, and 30 neurons), respectively. The training and testing loss curves are shown in Figure 9. While all three MLP networks eventually reached similar convergence levels in the later stages of training, the network with only one hidden layer exhibited slower loss reduction during the initial training phase. Considering both the network size and convergence speed, we opted for the network structure with two hidden layers.

**Figure 10:**
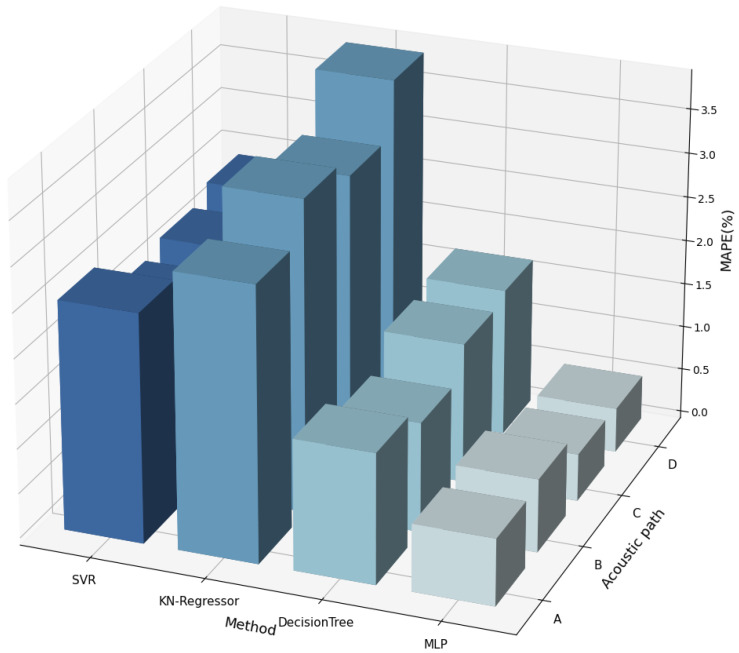
MAPE on different acoustic paths and methods.

Apart from this, we also complete the velocity prediction task using different methods, including Support Vector Regression (SVR), KNeighborsRegressor, and Decision Tree. In Figure 10, we compared the MAPE of these methods on different acoustic paths, proving that our method can always maintain the best prediction results. Further, the results of all acoustic paths are averaged and shown in Table 3. It can be observed that MLP achieves the highest prediction accuracy, which is about 1% higher than the other methods.

In addition, we conducted comparative experiments to prove the effectiveness of the loss function design. As shown in Table 4, the Mean Squared Error (MSE) loss is selected as the baseline. In this case, the MAPE is 2.13%. When using the relative error loss function ($loss_{1}$) and considering the mechanism of flow distribution ($loss_{2}$), respectively, the prediction accuracy of the model can be improved. By observing the data results, we can also find that when using $loss_{1}$, the model can maintain good prediction results even when the flow rate is small. When using $loss_{2}$, the predicted flow velocity of each vocal channel will be more consistent with the flow velocity distribution pattern of real flow data. This is not only in line with the original intention of the loss function design but also explains the source of performance improvement. The method we proposed combines the advantages of both aspects and has the best performance, improving 1.48% compared to the baseline.

## 4. Conclusions

In this work, an ultrasonic flow meter modeling method is proposed that combines data learning with mechanistic knowledge. In the data preprocessing phase, we employed diverse methods tailored to the specific characteristics of each dataset, considering both the data type and its significance in influencing the flow process. This meticulous approach ensures an optimal extraction of pertinent information while minimizing the effects of noise and irrelevant variables. In the loss function design, by classifying and discussing different working conditions, the specific statistical characteristics of flow velocity in real data under different working conditions can be better extracted. We incorporated the physical distribution law of flow velocity into the loss function as a constraining condition, aiming to align the model’s predictions as closely as possible with real acoustic path flow velocity distributions. Through extensive experimentation, we demonstrated the effectiveness of our approach in leveraging both mechanistic and data-driven features. The resultant model achieves high accuracy and interpretability, with an MAPE of only 0.65% on the test set, adequately meeting the requirements for ultrasonic flow meter modeling in practical production scenarios.

## Figures and Tables

**Table 1:** Dataset summary.

Feature	Range/Discription	Unit
Pipe diameter	100∼400	mm
Temperature	286∼300	K
Pressure	6.9∼9.3	MPa
Rectifier	Different brands	NULL
Collector pipe condition	Different inflow situations	NULL
Distance before rectification	0∼20	mm
Distance before flow meter	0∼20	mm
Standard flow rate	0.6∼30	m/s

**Table 2:** The relative error of five pieces of data on the test set.

Case	Error-A (%)	Error-B (%)	Error-C (%)	Error-D (%)
1	0.25	0.56	0.79	0.89
2	0.26	0.40	0.32	0.57
3	0.69	0.42	0.40	0.96
4	0.46	0.07	0.18	0.53
5	0.34	0.44	0.85	0.29

**Table 3:** Effectiveness of model selection.

Method	SVR	KN-Regressor	DecisionTree	MLP
MAPE (%)	2.33	3.42	1.48	0.65

**Table 4:** Effectiveness of loss function design.

Method	Baseline	loss-1	loss-2	loss-1 & loss-2
MAPE (%)	2.13	1.62	1.47	0.65

## Data Availability

The data that support the findings of this study contain proprietary information and cannot be made publicly available.
